# Perceptions of physicians in Saudi Arabia on the use of international clinical guidelines for managing primary insomnia

**DOI:** 10.1371/journal.pone.0220960

**Published:** 2019-08-09

**Authors:** Ali Dobia, Kath Ryan, Mohammed Abutaleb, Alexander Edwards

**Affiliations:** 1 Reading School of Pharmacy, University of Reading, Reading, United Kingdom; 2 General Directorate of Medical Services, Riyadh, Saudi Arabia; 3 Jazan Health Affairs, Ministry of Health, Jazan, Saudi Arabia; Medical University Graz, AUSTRIA

## Abstract

**Introduction:**

While there are no national clinical guidelines for managing primary insomnia in Saudi Arabia, there are also no published studies of physicians’ perceptions of and attitudes towards using international guidelines. The objective of this study was to explore the knowledge, perceptions, and attitudes of physicians practising in Saudi Arabia about using international guidelines for managing insomnia.

**Methods:**

A qualitative study using in-depth, face-to-face, and semi-structured interviews with 15 physicians held in July 2017 at a tertiary care hospital in Jazan, the distal south-western province in Saudi Arabia. Interviews were audio-recorded, transcribed verbatim, coded using the qualitative software NVivo11 and analysed thematically. Data saturation was assumed as no new understandings of the broad thematic issues were produced by the last three interviews.

**Results:**

Themes identified were: Knowledge, Resistance, Barriers and Facilitators. Participants acknowledged their lack of awareness of available guidelines and their lack of training and education about Cognitive Behavioural Therapy for Insomnia (CBT-I). They highlighted a lack of education for patients about insomnia and its treatment. Beliefs about dependence on hypnotics and the inappropriateness of international guidelines for Saudi Arabia inclined many to resist using them. Inability to document diagnosis and consultations due to limited time and lack of suitable electronic systems, lack of suitably trained practitioners for referral for CBT-I, and lack of accountability for practice were identified as key barriers to following international guidelines. Development of national guidelines was the most important facilitator suggested by participants.

**Conclusions:**

The health authorities in the government of the Kingdom of Saudi Arabia (KSA) should improve general public awareness about sleep disorders and provide focused training for specialists and technologists. Above all, KSA needs its own nationwide guidelines for treating sleep-disorders based on evidence-based clinical trials, consistent with its history, culture, socioeconomic conditions and traditions.

## Introduction

Clinical guidelines are known to reduce inconsistent prescribing practices [1]. Their purpose is to organize and provide the best available evidence to support clinical decision-making about the quality of care, patient outcomes and cost-effectiveness [2, 3]. While the number of guidelines for healthcare-use continues to grow, their practical implementation is often slow, complex, and unpredictable [4–6].

Although general practitioners have a largely favourable attitude to clinical guidelines, many do not follow them [7, 8]. The reasons for this are many and varied but a lack of applicability to patient needs is high among them. It appears not to matter whether the guidelines are prescriptive or proscriptive, whether they encourage or discourage certain types of behaviour or treatment; physicians are reluctant to follow them [9]. Nevertheless, non-adherence can lead to suboptimal or even inadequate treatments, and to unnecessary diagnostics [10].

Most methods used to change clinical practice have been based on beliefs rather than on scientific evidence [11]. To improve physician’s adherence to clinical guidelines, therefore, investigating knowledge, beliefs, and the presence of barriers and facilitators to following them is crucial [11, 12]. Many functional barriers to guideline-use have been identified, such as the practitioner’s limited expertise, the patient’s awareness of the drugs, and the social, cultural and organizational context in which the treatment occurs [11–15]. These barriers can be classified into personal, guideline-related, and external factors. A systematic review by Cabana and colleagues (1999) of barriers to physician adherence included 76 studies. The authors developed a framework in which barriers were classified into three categories: those related to the physician’s knowledge, (e.g., lack of awareness and lack of familiarity), those that influenced the physician’s attitude (e.g., lack of agreement and lack of motivation) and those related to behaviour (e.g., patient preferences, guideline characteristics and physician’s lack of time) [13]. In a more recent study (2009) that focused on guideline recommendations, the barriers most often identified were: the physician’s lack of agreement with the recommendations due to limited applicability or lack of evidence (68% of key recommendations), environmental factors such as organisational constraints (52%), lack of knowledge regarding guideline recommendations (46%), and factors such as unclear or ambiguous recommendations (43%) [16].

Healthcare authorities would benefit from understanding the perceptions of and attitudes towards the use or non-use of clinical guidelines by physicians. A recent qualitative study of 46 Spanish physicians, coordinated in six discussion groups, revealed two principal factors that influenced their perceptions of clinical guidelines: “knowledge” and a sense of “usefulness”. These were related to other factors, including confidence in guidelines, usability, accessibility, ease of dissemination, and guideline formats [17].

There is little qualitative research in the Middle East about the knowledge, perceptions and attitudes of general practitioners towards clinical practice guidelines (CPGs) that can help to facilitate implementation of good practice. A recent study in the United Arab Emirates, found that practitioners had a positive attitude towards CPGs and welcomed the use of evidence-based practice that is supported by electronic medical records, persistent quality monitoring and structured care programmes [18]. Participants, however, also expressed negative attitudes towards impractical guidelines, inconsistent recommendations among guidelines and the possibility of ‘changing evidence’ [18].

In Saudi Arabia, the few quantitative studies [19, 20] that have been conducted, found that practitioners had positive attitudes towards and were satisfied with CPGs. This was because of the potential of CPGs to reduce risk and variation in healthcare practice, improve safety, and be useful sources of advice for patients. A cross sectional survey conducted at the King Khalid University Hospital revealed that 99% of practitioners thought that CPGs were good tools for clinical practice in that they increased safety and uniformity of practice, thereby decreasing risk. In addition, 98% had confidence in well-developed guidelines especially for the management of diabetic keto-acidosis [19]. Almazrou and Alnaim (2018), in Riyadh, Saudi Arabia, found that 89% of physicians used clinical guidelines in the management of their patients either because they were based on evidence (84%) or because it was a requirement by the institutions where they work (40%) [21]. The main barriers against the implementation of clinical guidelines in the region were the lack of awareness of the guideline and knowledge of its use and application [21]. Also, a self-administered questionnaire conducted in Riyadh found that 40% of physicians did not consider sleep disorders a distinct specialty [22]. The researcher reported poor recognition of some serious consequences of sleep disorder and a lack in education and training among these physicians. Only 15% of them had attended lectures about sleep disorders during their postgraduate training or practice [22].

Insomnia is one of the most prevalent diseases in Saudi Arabia. It has been found to affect up to 78% of the population and the level of services provided in the country are below the level of services provided in other developed countries even though there have been improvements in these services since 2005 [23,24]. Benzodiazepines are also reported to be some of the most abused drugs among the youth in Saudi Arabia [25]. Accordingly, sleep medicine is considered to be a modern specialty in Saudi Arabia and it is being expanded due to the increasing demand for specialized practitioners to provide accurate diagnosis and appropriate management for sleep disorders including insomnia [24].

In the absence of national Saudi guidelines for treating primary insomnia, previous work has shown that many physicians follow (or at least consult) United States (US) guidelines, which the Saudi Ministry of Health (MOH) considers to be best practice [26, 27]. An audit, however, of medical records of patients who were prescribed benzodiazepines (BZDs) or Z-drugs (from April 2012 to March 2017 in the same institution as the interview study reported here) compared practice with US guidelines and found that physicians did not actually follow US or international guidelines [28]. Imperatively, further research is needed into the factors that guide diagnostic and treatment practices in Saudi Arabia. Therefore, in this paper, drawing on what participants had to say about their current knowledge and practices, we address the question: What are the perceptions, attitudes and knowledge of physicians in KSA towards international guidelines for managing primary insomnia?

## Methods

The study was conducted in a public tertiary care hospital located in Jazan Province, the distal southwestern region in Saudi Arabia. A purposive sample of physicians who were prescribers of BZDs or Z-drugs for insomnia between 2012 and 2017 was recruited. Potential participants were identified by the lead pharmacist, and provided with an invitation pack, containing an Invitation Letter and Information Sheet. Two potential participants chose not to proceed because they lacked adequate knowledge about the study subject. The researcher (AD) continued inviting physicians until the required number of 15 was reached. An interview guide, designed to encourage participants to talk in a conversational manner about their experiences, was developed based on the literature and findings from a previous audit study conducted in the same hospital [28]. The interview questions were piloted with one physician prior to conducting the study.

Face-to-face, in-depth, semi-structured interviews with AD were digitally audio-recorded with the physicians’ permission. Participants were interviewed in English using open questions open-ended such as “How do you manage patients with insomnia?”, “Which guidelines do you follow when prescribing for patients with insomnia?” and “Where did you learn about the guidelines?” Follow-up questions arose from the participant’s answers. Probing questions elicited further information if and when required. Participants were asked to reflect upon some of the findings from the previous audit [28].

Interviews were transcribed verbatim, anonymized, and entered into the qualitative software NVivo 11 (QSR International) for initial coding. The researchers met regularly to discuss the developing coding schema and resolve discrepancies to reduce the possibility of researcher bias. Data were then coded by AD using an inductive approach and analysed using thematic analysis [29]. The interviews were read and re-read and material relevant to each code was collated and relevant codes combined into potential themes and sub-themes [29]. The codes were read against each theme to ensure a coherent pattern and the same process was followed for the whole data set [29]. Verbatim extracts illustrating the themes were selected for reporting in this paper. Ethical guidance (approval number 17/15) was obtained from the University of Reading Ethics Committee (UREC) and from the Research Ethics Committee (REC) at the General Directorate of Jazan Health Affairs, MOH, KSA.

## Results

Fifteen physicians, who were prescribing BZDs and Z-drugs between April 2012 and March 2017, took part in interviews lasting 18–50 minutes. Table 1 shows selected demographic details of the participants.

**Table 1:** Demographic details of participants.

Variables–Number
**Gender** •Male: 14 •Female: 1
**Specialties** •Psychiatry with training in the field of sleep medicine (SM): 3 •Psychiatry: 5 •Neurology: 2 •Family medicine: 4 •Internal medicine: 1
**Professional status** •Consultant: 7 •Specialist: 6 •Resident: 2
**Years of experience** •<2 years: 2 •3–5 years: 2 •6–10 years: 4 •>10 years: 7
**Nationality** •Saudi Arabian: 7 •Egyptian: 5 •Sudanese: 2 •Syrian: 1
**Place of education and training** •Inside Saudi Arabia: 6 •Canada: 1 •Egypt: 5 •Sudan: 2 •Syria: 1

**Fig 1:**
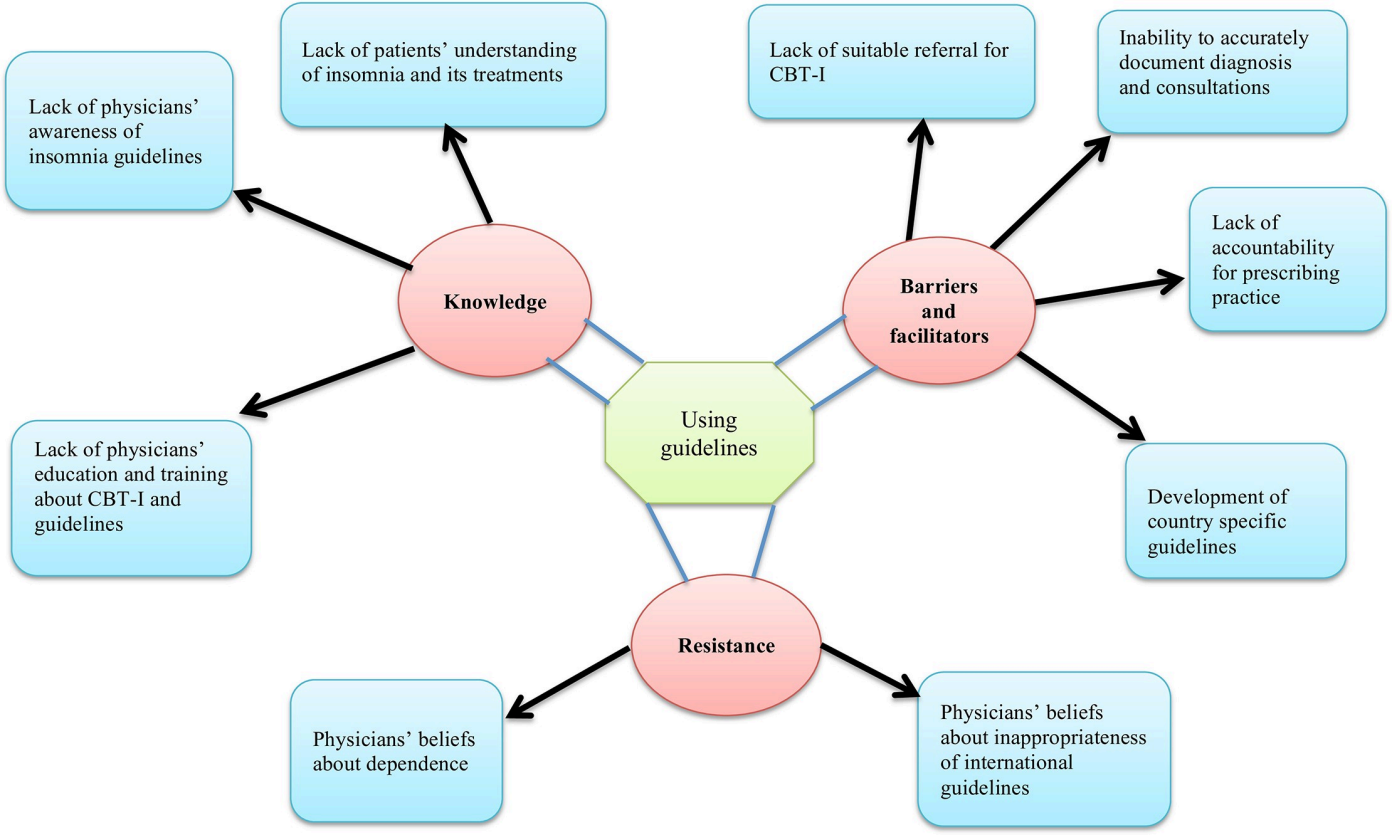
Analytical themes and subthemes.

Three principal themes (illustrated in Fig 1) were found from the interview data: knowledge, resistance, and barriers and facilitators. Sub-themes of knowledge encompassed lack of physician awareness of insomnia guidelines, lack of physician education and training of CBT-I and lack of patients’ understanding of insomnia and associated treatments. Sub-themes of resistance comprised beliefs of physicians about dependence and the inappropriateness of international guidelines to be implemented in their current care provision. Barrier sub-themes included inability to accurately document diagnosis and consultations, lack of CBT practitioners or lack of suitable referral for CBT-I, and lack of accountability for practice. Development of local guidelines for management of insomnia was the only facilitator. Themes and subthemes with illustrative quotations from participants (in italics) are presented below.

### Knowledge

Issues related to both physicians’ and patients’ knowledge of primary insomnia and appropriate treatments were identified through three subthemes.

#### Lack of physicians’ awareness of insomnia guidelines

When participants were asked about international guidelines for treating insomnia, some acknowledged that they knew little or nothing about them. They usually focus, they said, on their specialty, managing patients with insomnia according to what they know or had learned from colleagues.

*No fixed international or national guidelines applied, at least for me. I have never seen the guidelines*. *(Psychiatrist >10 years’ experience)*

*I don’t know if there is a specific guideline for insomnia … we depend on general advice about drugs that cause sleepiness, either as a side effect or as a target to treat sleep disorders or insomnia per se*. *(Neurologist>10 years’ experience)*

Some participants believed that such practices are common and that, while they might be unaware of specific guidelines for treating insomnia, they follow them nonetheless.

Maybe I am applying (the guidelines) without knowing. (Psychiatrist 6–10 years’ experience)

*If you are dealing with psychiatrists maybe they have specific guidelines to follow*, *but for non-psychiatrists I think we follow the general rules, you know, which usually come from the guidelines. They are not memorized, though; they make it as a subconscious practice but not according to the specific guidelines. (Neurologist>10 years’ experience)*

#### Lack of physicians’ education and training about CBT-I and guidelines

When asked why they did not follow US guidelines for using CBT-I as a first-line treatment, most participants said that they lacked appropriate education and training.

*We don’t have trained persons who can give Cognitive Behavioural Therapy to the patients*. *I think only a psychiatric hospital has that. (Family medicine <2 years’ experience)*

*I don’t know what you mean by this CBT or Cognitive Behavioural Therapy*. *(Neurologist>10 years’ experience)*

Asked whether they had a good knowledge about the guidelines and followed them for treating insomnia, some participants said that the health ministry should provide obligatory courses for training and encourage doctors to attend.

*I think “No” and the solution to this problem is to make courses and training for doctors*. *The training can be in psychiatric hospitals and to be announced for doctors in every hospital that you must attend or it would be better for you to attend this course for two or three days and to reinforce those doctors if they attend by certificates or continuing medical education hours (Psychiatrist 6–10 years’ experience)*.

#### Lack of patients’ understanding of insomnia and its treatment

Participants discussed their expectations about patients in KSA in terms of their understanding of insomnia, the side effects of medications, and the patient’s willingness to be referred to psychiatric hospitals for appropriate treatment. Physicians said that KSA patients are insufficiently informed to accept, for example, CBT-I treatments and/or they are unwilling to visit doctors without receiving prescription medicines.

*Some people, especially in our area, are not educated, so because you are a doctor*, *you should treat with pharmacological treatments. (Psychiatrist trained in SM 3–5 years’ experience)*

Stigma related to visiting a psychiatric hospital, even for insomnia, is well known in KSA. For this reason, many participants said that they cannot be sure that patients will attend follow-up appointments or agree to CBT-I treatment in a psychiatric hospital.

*Many patients who come to the clinic are still having the stigma. Even if he has insomnia and someone advises him to go to a psychiatric clinic, he is very reluctant to come to the clinic because, once he goes to the psychiatric clinic that means he is insane*. *So, the stigma of a psychiatric disorder including sleep disorders is still there, and even the doctor suffers a lot to explain psych-education. (Psychiatrist 6–10 years’ experience)*

Many participants were reluctant to prescribe BZDs or Z-drugs as a first-line pharmacological treatment for KSA patients, believing that many patients are insufficiently educated to stop taking the medication once they start. They thought that risks and side effects should be weighed against benefits, and BZDs or Z-drugs given only to well-informed patients.

*Yes, in many cases I write benzodiazepines for short periods of time, but the patients must be highly educated. I must be sure that he is not (inclined to) substance abuse, so I write. I don’t hesitate to write benzodiazepines for this patient*. *(Psychiatrist <2 years’ experience)*

*Our patients don’t read about the problem, about the medication itself, they don’t know about side effects. This is one of the major dilemmas in our practice*. *(Family medicine 6–10 years’ experience)*

### Resistance

Because of specific local challenges, such as culture and uneducated patients, some physicians resisted applying international guidelines. Reasons for resistance were identified through two subthemes.

#### Physicians’ beliefs about dependence

One criterion most physicians disagreed with was prescribing BZDs or Z-drugs as a first-line pharmacological treatment for patients with insomnia. Most said that they prefer not to prescribe them to avoid their risk and side effects. For example:

*I prefer the other medications [meaning anti-histamine with sedative effect*, *antidepressants or antipsychotics] because of fear of dependence. (Psychiatrist 6–10 years’ experience)*

*Last choice is BZDs and non-BZDs group because I am fearful of the dependence of these medications*, *especially here in our region. (Psychiatrist 6–10 years’ experience)*

A few participants, fearing patients’ dependence, believed that BZDs and Z-drugs should not be prescribed at all, even in the short term.

*I start believing do not give it at all. Within one or two days, okay, people will get used to that even if they are not truly addicted but it is a very cheap and fast and perfect solution for the insomnia, that’s what I believe*. *(Psychiatrist >10 years’ experience)*

Some participants held other views, believing that the drugs can be prescribed safely for short periods, but as mentioned above, only to well informed or highly educated patients who are unlikely to become dependent on them.

*We are afraid of tolerance and dependence… our patients are not educated well to avoid this… poor education is a problem in communicating with patients*. *(Psychiatrist <2 years’ experience)*

#### Physicians’ beliefs about the inappropriateness of international guidelines

Almost all participants had concerns about the suitability and applicability of international guidelines to their practice. The physicians spoke often of the difficulty in applying the guidelines to specific patients because of differences in culture, religion, and social norms.

*I think the guidelines are like, how can I say it, not applicable in our countries*. *(Psychiatrist <2 years’ experience)*

*In modifying, as I told you, I think there is some cultural, some genetics, some differences between our regions and western. So*, *their guidelines are slightly different and do not give such results which we are reading about. (Psychiatrist 6–10 years’ experience)*

### Barriers and facilitators

Participants discussed barriers that hinder them from following international guidelines for treating insomnia. Analysis identified three principal barriers.

#### Inability to accurately document diagnosis and consultations

Many participants raised the issue of poor hospital documentation. They believed that guideline recommendations cannot be followed because the recording system is flawed. They would not prescribe a recommended medication with known risks and side effects without a robust documenting system to monitor patients and to prevent them from duplicating their medication. It is still too easy, in the absence of adequate documentation, for patients to visit different clinics, even at the same hospital, to get the same medications.

*Maybe the problem is with* the *hospital system because it is dangerous to start medications without documenting the cause because maybe the next time you are not the one who will treat the patient*. *We have problems with documentation*. *If we start a patient with benzo he will continue forever on benzo because changing doctors and no documentation for how long he was using this medication and what was the cause*. *(Psychiatrist trained in SM 3–5 years’ experience)*

Even though the physicians acknowledged the importance of documentation, and agreed that it is a pressing issue, most were reluctant to complete the necessary documentation themselves. Workload, time-limits and difficulties of using the computer/record system were the principal barriers. They suggested that documentation should be carried out by others, possibly by trained clerical staff.

*Only few words (are) sometimes not efficient in the documentation. So, I think if there is a secretary or another person who can do this work, [it] will be helpful for the documentation and feedback*. *(Psychiatrist 6–10 years’ experience)*

*We’re supposed to do that. Frankly speaking not everyone is doing documentation including myself, of course, because of the tightness during the clinic especially, you know, if you have 25 patients within three hours and in the last one year we are dependent on the computer, so no more hand written so, again, there are some difficulties to type in the computer and sometimes the network is slow*. *(Neurologist>10 years’ experience)*

#### Lack of suitable referral for CBT-I

Participants raised the issue of the lack of appropriately trained health professionals, to whom to refer patients, which forces physicians to start the second line treatment immediately.

*For CBT, we don’t have expert professionals in CBT so usually they will skip to the next step in the guidelines*, *which is the medication (Psychiatrist trained in SM 3–5 years’ experience)*

We don’t have one good place for psychotherapy (Psychiatrist >10 years’ experience)

#### Lack of accountability for prescribing practice

It is also clear from the analysis that many instances of physician malpractice occur in prescribing and treating patients with insomnia. Such treatments endanger not only patients’ health but also their lives. Some participants believed that physicians and other professionals should be held accountable for the consequences of their treatments, particularly when they do not conform to standard practice.

Some hospitals have no strict system for prescribing medications. (Psychiatrist trained in SM >10 years’ experience)

*Hospital issues, maybe there is no good system [for] the doctors [to follow]*. *I think these are the most things I can explain*. *No clear rules in the hospital that require the doctors to see [follow-up] their patients. (Family medicine>10 years’ experience)*

*I think more and more malpractice and abuse for these medications especially in private hospitals. Some patients come to my clinic making quarrels with me because I am giving him only 10 tabs of Alprazolam and arguing with me and say I am going to that private hospital in Jeddah and I am receiving three boxes of Xanax. He is receiving about 300 tablets from private hospitals so he is shouting with me in the clinic to [prescribe] like the private hospitals*. *(Psychiatrist 6–10 years’ experience)*

#### Development of country specific guidelines

All participants expressed the need for guidelines to be developed and followed by all physicians in KSA hospitals. Such guidelines must align not only with the patients’ needs but also with the history, culture, social practices and socioeconomic conditions of KSA.

*I think the guideline is important. Patients are different from country to country, according to culture*, *according to education. Many factors are affecting patients and affecting our selection of drugs to our patients. (Psychiatrist <2 years’ experience)*

Country specific guidelines would help physicians diagnose and treat insomnia patients, many of whom present with symptoms related to cultural behaviours (for example the common use of Khat, a stimulant or amphetamine-like plant from Jazan Province), and have different treatment expectations from patients in other countries. Social barriers and other factors specific to KSA might affect the treatment of insomnia and need to be incorporated into country specific guidelines.

*Those who are using drugs like (Khat) or (Amphetamine) are definitely have insomnia and will seek help. They will [shop around] and try to get benzodiazepines. If they could not get it they will try to get another medication, which can induce sleep. [Our] culture is different [from other countries]*. *(Psychiatrist >10 years’ experience)*

Local guidelines would, to some extent, protect physicians if their practices were assessed, for example:

*Following our guidelines*, *it is not necessarily just aiming to give proper treatment but also to protect you and also the patient can go back to those guidelines and get convinced that the doctor is following the right guidelines. (Psychiatrist trained in SM >10 years’ experience)*

## Discussion

Physicians revealed that their current practices and choices on whether or not to use available international clinical guidelines when treating primary insomnia depended on knowledge of and resistance to guidelines, the presence of barriers limiting their use, and the lack of facilitators to encourage adherence to the stipulated guidelines. Although some of the themes identified in this study are similar to those discussed in previous studies [16–18], no research, to the best of the authors’ knowledge, has identified physicians’ resistance to implementing international guidelines as a principal theme.

Some of the physicians recruited for this study, especially those who had more than 10 years of experience, admitted that they were unaware of international guidelines for insomnia treatment and that they normally focused on their specialty, treating patients based on the clinical knowledge and skills they acquired at college or from their colleagues. This finding agrees with research conducted in Estonia by Pille Taba (2012), which found that physicians practicing for over 25 years or those who treat patients in an outpatient setting find it difficult to use guidelines. This is because their prescribing practices are based on personal experience and how patients react towards certain prescribed drugs [30].

Several physicians acknowledged that they lacked training in and education about sleep disorders and associated treatment, which affected their practices. They said that they cannot provide CBT-I for their patients because they lack knowledge of and training in such treatments and do not have trained colleagues (eg. psychologists) to whom to refer patients. This finding supports studies conducted by Almeneessier & BaHammam (2017) who found that KSA lacks the education and training to help insomnia specialists to practice in the field [31], and a World Health Organisation mental health atlas KSA country profile (2014) that reported 1.38 psychologists per 100,000 population in KSA (population: 32 million) [32].

One factor that many physicians underscored was the patient’s education about insomnia and its treatments and how it influenced, either positively or negatively, their (the participant’s) clinical decision about following guidelines. Some participants stated that patients have been known to harass specialists for the medications they want, disregarding professional clinical advice, in contrast to what is happening in Europe, where many patients specifically choose psychological help [33]. Participants said that poor education of their patients resulted in feelings of being stigmatized if they were referred to a psychiatric hospital for non-pharmacological treatment or counselling. There is, additionally, the problem of the patient’s health literacy, since many patients have little or no idea about the effectiveness and level of risk of dependence of some pharmacological treatments. In any case, patients can always seek a more compliant doctor, who will prescribe the drugs they want but which might not be in their best healthcare interests [34].

It is also clear that many physicians are reluctant to prescribe BZDs for their patients, even though these drugs are recommended in many international guidelines. This dated attitude towards using such medications affects best clinical practice and explains why physicians often use other medications such as zolpidem, antidepressants or antipsychotics. This finding agrees with a study conducted in Britain by Siriwardena et al., (2006), which indicated that general practitioners often prefer prescribing Z-drugs over BZDs, believing them to be more effective and safer, which is unsupported by the evidence [35]. In fact, this belief might be a cause for resistance to international guidelines about hypnotics. According to Siriwardena et al., (2006), the problem has persisted for several years with most studies finding that physicians decide which medications to use according to their understanding of the medication’s risk and side effects [35].

Generally, participants in the current study recognized the value of guidelines but they also believed that adherence must be flexible and adjusted according to the patient’s educational status and to national contexts. The physicians maintained that international guidelines were valuable only if adapted for regional use. Inevitably, this leads to considerable resistance and non-adherence because cultures, religions and social values often diverge. We need to understand that countries are as different from one another as patients are from each other, and that those differences need to be considered. A study by Lugtenburg (2016) with Dutch general practitioners indicated that the lack of applicability to local conditions is the principal barrier to international guidelines being followed [16].

The need for a robust records system is also required to provide comprehensive documentation of patient care. Physicians indicated that their documentation system has several loopholes and that they tend to forego documentation of administrative issues, which contributes to poor records yet, paradoxically, they said that documentation is crucial for drug prescription purposes. This argument correlates with systematic review findings of Hasanain et al., (2014), that although the KSA government introduced the Electronic Medical Record [19] in 1988, the documentation system faced the challenges of software complexity, cost overruns, privacy concerns, inadequate uniform standards, and inadequate vendor maintenance and support [36]. Insomnia medications such as BZDs or Z-drugs are two prescriptive classes of drugs that require adequate documentation to ensure that patients are well monitored. The current study identified the lack of a user-friendly, reliable record system as a barrier to optimal patient care since many physicians were reluctant to prescribe medications such as BZDs or Z-drugs without adequate patient records.

Lack of accountability is still another challenge to consider with regard to the use of clinical guidelines [37]. Most KSA hospitals, especially private hospitals, do not have a reliable or clear system to track physician behaviour, which would encourage best practice and decrease instances of malpractice. The lack of accountability might lead to negligence and weak performance of practitioners and imperil patients’ health and even their lives. Birrenbach et al. (2016) assessed the attitudes of physicians in Switzerland towards clinical guidelines and revealed that they believed that clinical guidelines were likely to improve quality of care and decrease malpractice [38]. Therefore, the MOH and other health sectors in Saudi Arabia should consider mandatory implementation of clinical guidelines in their hospitals to make healthcare professionals accountable for their practices. Moreover, implementation should be followed up with evaluation and monitoring of physicians’ behaviours to ensure best practice.

Farquhar et al., (2002) in their systematic review revealed that most clinicians in Western countries viewed clinical practice guidelines as useful, cost-saving, educational and likely to improve quality of care. Some clinicians also perceived clinical guidelines as impractical for individualised care, potentially increasing litigation or disciplinary action and restricting clinician’s autonomy [7].

Physicians in our study in Saudi Arabia paralleled the positive perceptions about guidelines of their Western counterparts but many claimed that international guidelines are inapplicable in KSA because of the different cultural, religious and social norms. They believed that physicians and other professionals should be held accountable for the consequences of their practices, however, there was no mention of litigation, limited clinician autonomy or costs of health care.

This study is the first of its kind to explore physicians’ perceptions about treating insomnia in the KSA. The semi-structured interview enabled participants to take the lead and to discuss issues of importance to them. Data was analysed thematically which enabled the identification of key themes documenting the views of KSA physicians about their prescribing practices and attitudes towards international guidelines, giving them voice for the first time.

Hennick et al., (2016) stated that code saturation is usually reached after nine interviews, sufficient for the identification of broad thematic issues or the development of a survey instrument, but 16–24 interviews are needed to reach meaning saturation [39]. This study has produced a rich description of a purposive sample of Saudi physicians’ attitudes towards the use of guidelines, with several examples of variation in each code and theme, which assisted the presentation of data that was appropriate and adequate. Since no new understandings were discerned from the last three interviews, data saturation was assumed to have occurred.

Although the study was conducted in a single site and interviewed only 15 physicians, which can be considered as limitations, it reflects the common structure of the health system in the KSA. Most provinces have one central tertiary care hospital that receives referrals from general hospitals and their nearby primary care centres. The study was conducted in a tertiary hospital in KSA belonging to the government sector, and reflects practices for more than 60% of the population countrywide. We, therefore, expect that findings are transferable and can be extrapolated to similar contexts.

## Conclusion

Practice in this hospital and probably across the KSA is below internationally accepted standards and urgent action is required. The current situation does not encourage physicians to follow international guidelines, and therefore many ignore them. There are also cultural influences, chiefly in the form of beliefs and attitudes that affect both patients and physicians.

The KSA medical community should be familiarised with sleep disorders and their treatments and be held accountable for their prescribing practices. Medical practitioners should be able to avail themselves of all possible treatment regimens for their patients, including CBT-I, which should be provided by psychologists in non-psychiatric hospital settings. The government of KSA should enhance awareness in the general public about sleep hygiene and disorders. The MOH should establish a robust electronic system linking all hospitals and primary health care centres, and encourage documentation. They should also regulate prescribing behaviour and hold professionals to account for their practices. Above all, KSA needs its own evidence-based nationwide guidelines for treating sleep disorders according to its culture and socioeconomic conditions and traditions.
